# Clinical outcomes of Impella 5+ in acute myocardial infarction-related cardiogenic shock: a retrospective single-center study

**DOI:** 10.3389/fcvm.2026.1840979

**Published:** 2026-07-21

**Authors:** Giulia Maj, Alberto Piermartiri, Serena Querio, Marta Bandini, Marco Lombardi, Edoardo Elia, Serena Penpa, Riccardo Mazzucco, Federico Pappalardo, Corrado Cavozza, Alina Gallo, Andrea Audo, Giuseppe Patti, Gioel Gabrio Secco

**Affiliations:** 1 Cardiothoracic and Vascular Anesthesia and Intensive Care, AOU SS Antonio e Biagio e Cesare Arrigo, Alessandria, Italy; 2 Cardiology Department, IRCCS Policlinico Sant’Orsola, University Hospital of Bologna, Bologna, Italy; 3 Cardiothoracic Intensive Care Department, Royal Papworth Hospital, Cambridge, United Kingdom; 4 Interventional Cardiology Department and Cardiac Surgery Unit, AOU SS Antonio e Biagio e Cesare Arrigo, Alessandria, Italy; 5 Research and Innovation Department (DAIRI), University Hospital SS Antonio e Biagio e Cesare Arrigo, Alessandria, Italy; 6 Kore University, Enna, Italy; 7 Policlinico Centro Cuore GB Morgagni, Catania, Italy; 8 Cardiac Surgery Department, AOU SS Antonio e Biagio e Cesare Arrigo, Alessandria, Italy; 9 Department of Traslational Medicine, Univeristy of Eastern Piedmont, Novara, Italy

**Keywords:** cardiogenic shock, heart failure, impella, impella 5+, ischemic, mechanical circulatory (MCS) support

## Abstract

**Background:**

Temporary mechanical circulatory support (t-MCS) with Impella CP has recently attracted considerable interest as a therapeutic option for cardiogenic shock (CS) complicating acute myocardial infarction (AMI). In contrast, evidence regarding the use of the larger Impella 5+ (5.0/5.5) in this clinical setting remains limited. This single-centre retrospective study aimed to evaluate the clinical impact of Impella 5+ in patients with AMI-related cardiogenic shock (AMI-CS)

**Methods and results:**

Between January 2022 and August 2024, 28 patients referred for AMI-CS were treated with Impella 5+. 11 patients were classified as SCAI C, 11 as SCAI D and 6 as SCAI E. On admission, median inotropic score was 10.5 and median left ventricular ejection fraction was 17.5%. 12 patients required concomitant ECMO support (ECPella). 19 patients were escalated from IABPM to Impella 5+. The median length of support was 8 days, 2 patients (7%) received the support for more than 30 days. 1 patient experienced a major bleeding complication. The overall survival during index hospitalization was 79%: 3 patients were bridged to a durable left ventricular assist device (LVAD) and 2 received heart transplantation (HTx).

**Conclusions:**

Impella 5+ appears to be a safe and effective option in AMI-CS, associated with improved survival and a relatively low incidence of vascular and non-vascular complications. Its advanced features broaden the therapeutic potential of microaxial flow pumps in this high-risk setting.

## Graphical Abstract

**Figure:**
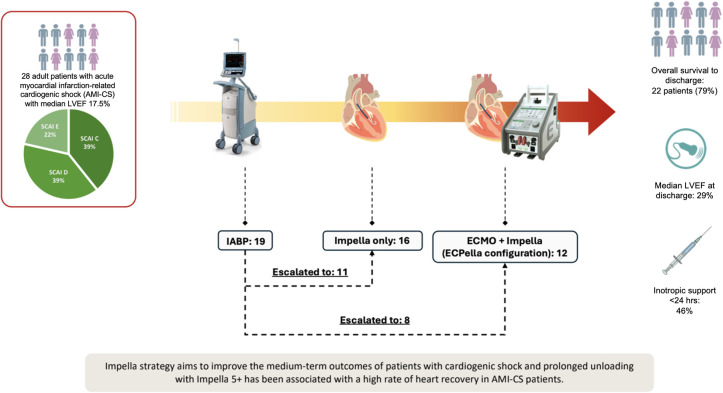

## Introduction

Acute coronary syndromes (ACS) account for a significant cause of morbidity and mortality, especially when complicated with cardiogenic shock (CS) (1, 2).

Despite the widespread use of primary percutaneous coronary intervention (pPCI) and advances in critical care management, early mortality of CS remains high at 30% to 50% over the past 2 decades (3, 4).

Temporary mechanical circulatory support (t-MCS) with Impella CP has recently been incorporated into clinical guidelines. Impella devices (Abiomed, Danvers, MA) have been shown to rapidly reduce left ventricular wall stress, stroke work, and myocardial oxygen demand (MvO_2_), while increasing systemic mean arterial pressure (5, 6). Together, these effects contribute to hemodynamic stabilization and effective left ventricular unloading (7, 8). Notably, the Danish-German Cardiogenic Shock trial (DanGer Shock) demonstrated a survival benefit with Impella CP in a highly selected cohort of patients with STEMI-related CS, albeit at the cost of increased complications (9). Device selection, however, remains a key determinant of outcomes, as it must be tailored to the patient's clinical presentation, hemodynamic profile, and institutional expertise. In particular, some patients may require higher flow support and prolonged unloading to restore organ perfusion and enable cardiac recovery (10). The aim of the present study is to report our institutional experience with Impella 5+ in the treatment of AMI-related CS, focusing on efficacy and safety.

## Methods

### Study population and design

All consecutive patients admitted to our Intensive Care Unit (ICU) between January 2022 and August 2024 who received Impella 5.0/5.5 for acute myocardial infarction–related cardiogenic shock (AMI-CS) were included. Follow-up was extended to 12 months. The study was approved by the local ethics committee and conducted in accordance with the Declaration of Helsinki. Written informed consent was obtained from all participants. This study was conducted and reported in accordance with the Strengthening the Reporting of Observational Studies in Epidemiology (STROBE) guidelines (Supplementary Table S1).

### Data collection

Clinical data were retrieved from the electronic medical record system and the Alt-Shock registry, and stored in REDCap (Nashville, TN, USA) (Altshock-2, NCT04369573). Baseline characteristics included demographics, comorbidities, laboratory findings, severity of CS according to the Society for Cardiovascular Angiography and Interventions (SCAI) classification, extent of coronary artery disease, and type of revascularization. Hemodynamic and echocardiographic parameters were recorded at ICU admission. Inotropic support was assessed at baseline and after 24 h; the vasoactive inotropic score was calculated as follows: dopamine dose (μg/kg/min) + dobutamine dose (μg/kg/min) + [100 × epinephrine dose (μg/kg/min)] + [10 × milrinone dose (μg/kg/min)] + [10,000 × vasopressin dose (U/kg/min)] + [100 × norepinephrine dose (μg/kg/min)].

### Definitions

Heart recovery was defined as successful weaning from both inotropes and mechanical circulatory support (MCS) without requiring heart transplantation or durable LVAD implantation during the index hospitalization. Impella-related variables included device type, timing of implantation, procedural duration, and total support time.

### Clinical scenarios of Impella use

Patients were stratified according to three predefined scenarios:

1. first-line device in isolated left ventricular dysfunction;
2. escalation strategy in patients already supported with IABP and/or ECMO;
3. unloading strategy in patients supported with ECMO for eCPR (see graphical abstract).

### Study endpoints

The primary endpoint was survival to discharge. Secondary endpoints included length of hospital stay, complications during Impella support, rate of native heart recovery, and bridge to advanced therapies (durable LVAD and/or transplantation). The decision to escalate or de-escalate mechanical circulatory support was based on a multidisciplinary discussion involving intensive care physicians, cardiologists, and cardiac surgeons. Management decisions were guided by an integrated assessment of the patient's clinical status, haemodynamic profile, end-organ perfusion, biochemical markers, echocardiographic findings, and the potential for myocardial recovery or transition to advanced therapies.

### Statistical analysis

Categorical variables are presented as frequencies (percentages), and continuous variables as mean ± standard deviation (SD) or median with interquartile range (IQR), as appropriate. The distribution of continuous variables was assessed using the Shapiro–Wilk test. Comparisons were performed with Pearson's Chi-square test or Fisher's exact test for categorical variables, as appropriate. Repeated continuous measurements were compared using repeated-measures analysis of variance (ANOVA) for normally distributed variables and the Friedman test for non-normally distributed variables. Statistical analyses were conducted with R 4.4.2 (R project for statistical computing, Vienna, Austria). A two-sided *p*-value < 0.05 was considered statistically significant.

## Results

Between January 2022 and August 2024, 28 patients with AMI-CS were treated with Impella 5+: 11 (39%) with Impella 5.5 and 17 (61%) with Impella 5.0. The cohort was predominantly male (27 patients, 96%) with a mean age of 66 ± 8 years. At admission, 11 patients (39%) were classified as SCAI C, 11 (39%) as SCAI D, and 6 (21%) as SCAI E. Half of the study population was referred from spoke hospitals. Baseline demographics and clinical characteristics are summarized in Table 1. Nine patients presented after out-of-hospital cardiac arrest (OHCA), most commonly with ventricular fibrillation (8/9, 89%). The median inotropic score at the time of implantation was 10.5 (IQR: 5–23.48).

**Table 1:** Demographics and baseline clinical characteristics of the study population.

Variable	Overall (*n* = 28)
Demographic characteristics	
Age, years	67 ± 8.8
Male sex	27 (96)
Height, cm	175 (170–178.5)
BMI, kg/m^2^	25.6 (24.2–27.8)
Comorbidities	
Diabetes	10 (36)
Hypertension	13 (46)
Dyslipidemia	10 (36)
Chronic kidney disease	4 (14)
Asthma/COPD	3 (11)
Atrial fibrillation	2 (7)
Stroke	0
Presence of ICD/CRT	1 (4)
Pacemaker	0
History of myocardial infarction	10 (36)
Previous PCI	10 (36)
Previous CABG	4 (14)
NYHA functional class before admission	
I	18 (64)
II	10 (36)
III	0
IV	0
Rhythm of presentation	
Ventricular fibrillation	8 (89)
PEA	1 (11)
Low-flow (*n*)	9 (32)
Low-flow time (min)	11 ± 5.7
Mechanical chest compression	7 (78)
SCAI stage	
A	0
B	0
C	11 (39)
D	11 (39)
E	6 (21)
Baseline echocardiography	
Left ventricular ejection fraction, %	17.5 (15–25)
Left ventricular end-diastolic diameter, mm	66.5 (61.8–69.2)
Severe mitral regurgitation	13 (48)
Right ventricular dysfunction	8 (30)
Treatment before hospitalization	
ACEi/ARB/ARNI	9 (32)
Diuretics	7 (25)
MRA	5 (18)
Calcium-channel blockers	5 (18)
Sildenafil	1 (4)
Anti-arrhythmic	2 (7)
Statin	9 (32)
Aspirin	10 (36)
Dual antiplatelet therapy	1 (4)
Oral hypoglycaemic	6 (21)
Insulin	5 (18)
GLP-1 receptor agonists	1 (4)
SGLT2 inhibitors	3 (11)

Data are presented as mean ± standard deviation (SD), median [interquartile range (IQR)], or *n* (%), as appropriate.

ACEi, angiotensin-converting enzyme inhibitors; ARB, angiotensin-receptor blocker; ARNI, angiotensin-receptor/neprilysin-inhibitor; BMI, body mass index; CABG, coronary artery bypass graft; COPD, chronic obstructive pulmonary disease; CRT, cardiac resynchronization therapy; GLP1-inhibitors, glucagon-like peptide 1-receptor antagonists; ICD, implantable cardioverter defibrillator; MRA, mineralocorticoid receptor antagonist; NYHA, New York heart association; PEA, pulseless electrical activity; PCI, percutaneous coronary intervention; SCAI, society for cardiovascular angiography and interventions; SGLT2-i, sodium-glucose transport protein 2 inhibitors.

All patients had cardiogenic shock secondary to ACS, presenting as STEMI (19 patients, 68%) or high-risk NSTEMI (9 patients, 32%). The majority (75%) underwent culprit lesion revascularization on an emergent basis, most frequently involving the left anterior descending artery (LAD) (see Supplementary Table S2). Eleven patients (40%) had multivessel disease; in these cases, complete revascularization under Impella support was staged following hemodynamic stabilization. The median door-to-balloon time in STEMI patients was 120 min (Table 2). On admission, median left ventricular ejection fraction (LVEF) was 17.5 (IQR: 15–25); 13 patients (48%) had severe functional mitral regurgitation, and 8 (30%) had concomitant right ventricular dysfunction.

**Table 2:** Primary indication, cardiogenic shock aetiology, periprocedural results and mechanical circulatory support features.

Variable	Overall (*n* = 28)
Primary indication	
Cardiogenic shock	25 (89)
Cardiac arrest	9 (32)
Intrahospital cardiac arrest	7 (78)
Aetiology	
STEMI	19 (68)
NSTEMI	9 (32)
Door-to-balloon in STEMI (minutes)	120 (60–150)
Door-to-support (minutes)	230 (120–990)
Spoke-to-hub transfer	13 (46)
Mechanical circulatory support	
Impella 5.0	17 (61)
Impella 5.5	11 (39)
Right axillary artery access	27 (96)
Trans-aortic access	1 (4)
ECMO support	12 (43)
IABP before Impella	19 (68)
Escalation strategy	19 (68)
V-A ECMO (ECpella configuration)	8 (29)
Impella implantation length (minutes)	90 (60–120)
Periprocedural complications	
Any complication	2 (7)
Hemothorax	1 (50)
Access-site bleeding	1 (50)

Continuous variables are presented as mean ± standard deviation (SD) or median [interquartile range (IQR)], as appropriate. Categorical variables are presented as *n* (%).

ECMO, ExtraCorporeal membrane oxygenation; iNO, inhaled nitric oxide; ECPella, combined V-A ECMO and impella; IABP, intra-aortic balloon pump; NSTEMI, non-ST-elevation acute myocardial infarction; STEMI, ST-segment-elevation acute myocardial infarction.

Regarding device strategy, 12 patients (43%) were treated with ECpella due to severe CS (SCAI E) or OHCA, while 16 patients (57%) received Impella support alone. Nineteen patients (68%) were escalated from intra-aortic balloon pump (IABP). A pulmonary artery catheter was used in 16 patients (57%), predominantly in those presenting with advanced cardiogenic shock (SCAI stage D or E), to support haemodynamic assessment and guide management. The preferred access was the right axillary artery (27 patients, 96%), while one patient required a trans-innominate approach via mini-sternotomy.

**Figure 1:**
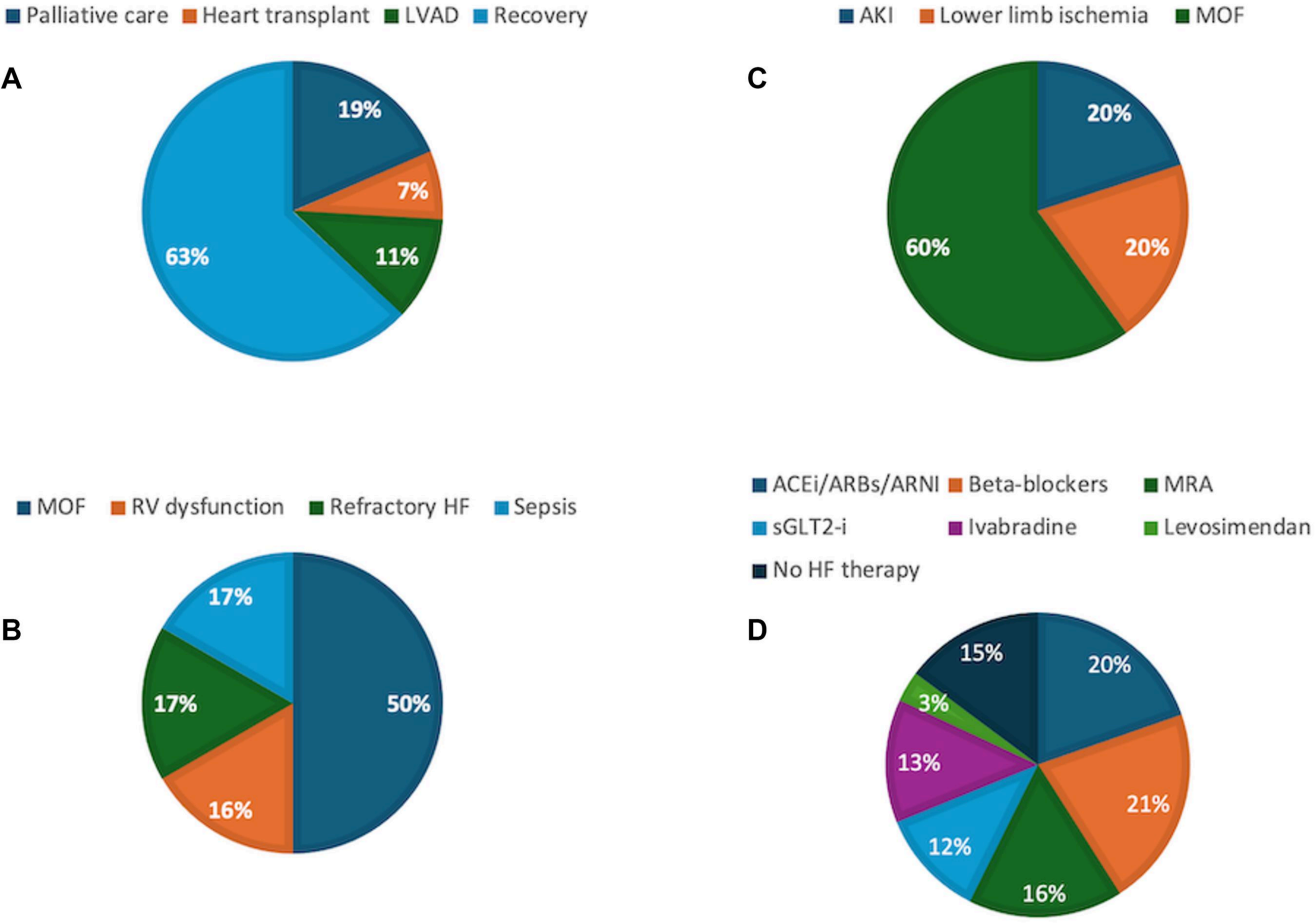
In-hospital clinical outcomes and future trajectory, post-operative complications and heart failure guidelines-directed medical therapy at MCS explantation. **(A)** In 17 patients (63%), Impella enabled cardiac recovery; 4 patients were successfully bridged to a heart replacement strategy [either as durable left ventricular assist device (LVAD) implantation or heart transplantation]. Heart recovery was defined as a patient successfully weaned off inotropes and/or mechanical circulatory support (MCS) for which a heart transplant or a long-term LVAD were not required during the index hospitalization. **(B,C)** Details about in-hospital mortality and complications. Multiple organ failure (MOF) was the most common cause of death (3 patients, 50%) and the most frequent post-operative complication. **(D)** After MCS explantation, in 85% of cases at least one heart failure drug was successfully restored, taking advantage of the combined effect of MCS on left ventricular unloading.

Overall hospital survival was 79%. Seventeen patients (60%) were successfully weaned off MCS with native heart recovery, while 5 patients (18%) required advanced therapies: 2 underwent urgent heart transplantation and 3 received durable LVAD implantation (Table 3, Figure 1A). At 12-month follow-up, 15 patients (67%) were alive. The median LVEF at Impella explantation was 29% (IQR: 20–35). Causes of death included multiple organ failure (3 patients, 50%), sepsis (1, 17%), refractory heart failure (1, 17%), and right ventricular dysfunction (1, 16%) (Figure 1B).

**Table 3:** Patients in-hospital outcomes.

Variable	Overall (*n* = 28)
Duration of mechanical circulatory support and hospital stay (days)	
ECMO duration	4.4 ± 1.4
IABP duration	2.7 ± 3.6
Impella support duration	8 (5.8–13.5)
Length of hospital stay	16.5 (8–25)
Postoperative complications	
Acute renal failure treated with CVVH	1 (10)
Lower limb ischemia	2 (40)
Multiorgan failure	3 (50)
Survived to discharge	22 (79)
Death	6 (21)
Causes of death	
Multiorgan failure	3 (50)
Refractory heart failure	1 (17)
Sepsis	1 (17)
Right ventricular dysfunction after VAD implantation	1 (16)
Palliative care	5 (19)
Heart transplant	2 (7)
Left ventricular assist device (LVAD)	3 (11)
Recovery	17 (63)
Left ventricular ejection fraction at Impella explantation, %	29 (20–35)
GDMT HF therapy at MCS explantation	
ACEi/ARB/ARNI	12 (42)
Beta-blocker	13 (46)
MRA	10 (35)
SGLT2 inhibitor	7 (25)
Ivabradine	8 (28)
Levosimendan	2 (7)
Comprehensive HF therapy^a^	5 (17)
No therapy	9 (32)

Continuous variables are presented as mean ± standard deviation (SD) or median [interquartile range (IQR)], as appropriate. Categorical variables are presented as *n* (%).

ACEi, angiotensin-converting enzyime inhibitors; ARB, angiotensin receptor blockers; ARNI, angiotensin receptor neprilysin inhibitor; CVVH, continuous veno-venous hemofiltration; ECMO, ExtraCorporeal membrane oxygenation; GDMT, guideline-directed medical therapy; HF, heart failure; IABP, intra-aortic balloon pump; LVAD, left ventricular assist device; MCS, mechanical circulatory support; MRA, mineralcorticoid receptor antagonists; SGLT2-i, sodium-glucose transport protein inhibitors.

a Comprehensive heart failure therapy including ACEi/ARBs/ARNI + beta-blockers + MRA + SGLT2-I at MCS explantation.

During hospitalization, 2 patients received adjunctive interventions (Supplementary Table S3): one underwent transcatheter mitral valve edge-to-edge repair (m-TEER), and another underwent combined mitral and aortic valve replacement with cardiac resynchronization therapy. Major complications included multiple organ failure (3 patients, 50%), lower limb ischemia (2, 40%), and acute kidney injury requiring continuous renal replacement therapy (1, 10%) (Figure 1C).

After Impella implantation, 12 patients (46%) were weaned off inotropes within 24 h (Supplementary Table S4). The median duration of Impella support was 8 days (IQR: 5.8−13.5), with 2 patients (7%) supported for >30 days. ECMO support, when used, lasted a mean of 4 ± 1.4 days, enabling early initiation of guideline-directed medical therapy (GDMT) and potential synergistic benefit with LV unloading (Figure 1D).

Device implantation was generally safe: only two major complications were observed—one case of access-site bleeding requiring surgical revision on day one and one case of hemothorax treated with surgical drainage.

Comparative laboratory analyses demonstrated significant reduction in lactate levels (*p* < 0.001). Hemodynamic stabilization was also achieved, with reductions in heart rate (median at ICU discharge: 80 bpm, *p* = 0.002) and significant improvements in mean arterial pressure (68 mmHg, *p* < 0.001), systolic blood pressure (100 mmHg, *p* < 0.001), and diastolic blood pressure (60 mmHg, *p* = 0.002). Detailed results are reported in Table 4.

**Table 4:** In-hospital comparative results for laboratory findings.

Variable	Pre	During Impella	Post	*P* value^a^
sCreat. (mg/dL)	1.18 (0.98–1.67)	1.26 (0.94–1.81)	1.43 (0.90–2.12)	0.490
PLTs	212 (186.5–256.5)	203 (129–243)	136 (95–195)	0.003
ALT (U/L)	72.5 (36–226)	132.5 (36.25–361.5)	36 (18.25–72.25)	0.001
Bilirubin (mg/dL)	0.8 (0.6–1.1)	0.9 (0.6–1.0)	1.0 (0.6–1.4)	0.448
Peak hs-Tn (ng/L)	45,758.5 (4,949.25–125,000)	81,228 (14,872.25–125,000)	941 (179.25–5,000)	<0.001
pH	7.40 (7.29–7.44)	7.41 (7.29–7.46)	7.41 (7.38–7.47)	0.698
Lactate (mg/dL)	2.5 (1.8–6.0)	3.6 (1.9–6.2)	1.1 (0.6–2.5)	0.002
SBP (mmHg)	90 (82.5–101)	85 (70–94)	100 (92.5–110)	0.001
DBP (mmHg)	50 (40.5–56.5)	49 (40–55)	60 (51–70)	0.002
MAP (mmHg)	63 (57–71.5)	57.5 (52.5–65.75)	68 (62.25–80.75)	<0.001
HR (beats/min)	100 (80–110)	95 (78.5–105)	80 (77–96.5)	0.002
CVP (cmH_2_O)	10.5 (8–13.5)	12 (8.75–13.25)	9 (8–10)	0.283

Continuous variables are presented as mean, median, range and standard deviation according to the frequency distribution.

a The *p*-values were set <0.05 and refer to comparisons among the three groups.

ALT, alanine aminotransferase; CVP, central venous pressure DBP, diastolic blood pressure; HR, heart rate; hs-Tn, high-sensitivity troponin; MAP, mean arterial pressure; PLTs, platelets count; sCreat., serum creatinine; SBP, systolic blood pressure.

## Discussion

In this retrospective, single-center study, we evaluated the impact of Impella 5.0 and 5.5 in patients with ACS-related cardiogenic shock (AMI-CS). Our results showed that 22 of 28 patients (79%) survived to hospital discharge, and 15 patients (68%) remained alive at 12 months. Importantly, a substantial proportion of the cohort presented with advanced shock (39% SCAI D and 21% SCAI E), and nearly one-third (32%) were referred after out-of-hospital cardiac arrest (OHCA), underscoring the severity of this population.

Survival in our study compares favorably with previous reports. Data from the Cardiogenic Shock Working Group (CSWG) showed a higher in-hospital mortality (45.2%) in AMI-CS patients supported with high-flow Impella compared to those with HF-related cardiogenic shock (HF-CS) (11). Similarly, in a multicenter analysis, Nakamura et al. reported lower 30-day mortality among patients treated with ECPella and Impella 5.0/5.5 compared with Impella 2.5/CP (35.6% vs. 56.8%) (12).

The advantages of Impella 5+ over lower-capacity devices or other MCS strategies are likely attributable to its ability to deliver higher flow (up to 5.5 L/min), providing robust hemodynamic support with greater reductions in myocardial oxygen consumption and more effective left ventricular unloading (13). In AMI-CS, where cardiac output deteriorates abruptly with rapid progression to multi-organ failure, early stabilization with high-flow support is crucial. In our cohort, this was evidenced by significant reductions in lactate levels (*p* < 0.001), alongside consistent improvements in hemodynamic parameters (heart rate, mean arterial pressure, systolic blood pressure). While lactate is not a direct marker of cardiac dysfunction, it reflects metabolic derangement and can dynamically capture disease severity and response to therapy, making it a potentially valuable prognostic and monitoring tool.

Notably, the incidence of complications in our study was relatively low. The DanGer Shock trial raised concerns regarding bleeding and vascular complications with Impella CP (moderate-to-severe bleeding 21.8%, limb ischemia 5.6%) (9), and Scherer et al. also reported an increased risk of bleeding with Impella compared to conventional treatment (14). Vascular complications have similarly been reported in patients receiving axillary Impella 5.5, particularly in the setting of AMI-CS (15). However, in our population, only one patient required surgical revision for access-site bleeding. This favorable safety profile may in part reflect the surgical axillary approach, which allows more controlled hemostasis, especially in patients requiring dual antiplatelet therapy (16).

To our knowledge, this is the first study to describe a complete revascularization strategy in STEMI-CS under Impella 5+ support. After culprit lesion revascularization, staged PCI of residual lesions was systematically performed once hemodynamic stability was achieved. This approach, combined with the hemodynamic benefits of Impella 5+, may represent a novel treatment paradigm in AMI-CS, creating a platform for comprehensive revascularization and supporting native heart recovery.

Finally, our study reflects a real-world cohort, including patients with heterogeneous presentations (OHCA, SCAI C–E) and a high proportion referred from spoke centers with limited MCS availability. Initial support strategies varied (e.g., ECMO for cardiac arrest, IABP in PCI-only centers), but escalation to Impella 5+ was ultimately employed to provide sustained, high-capacity, hemocompatible support (17). This underscores the potential role of Impella 5+ not only as a primary strategy but also as part of a stepwise escalation approach in AMI-CS management, complementing Impella CP use during PCI when additional support is required.

## Limitations

This study has several limitations. First, its single-center, retrospective, and observational design inherently limits causal inference and increases the risk of selection bias. Second, the relatively small sample size reduces the statistical power of our findings and may limit their generalizability. Third, the study population was predominantly composed of male patients, limiting the generalizability of these findings to female patients. Finally, the absence of a randomized control group precludes direct comparison with alternative mechanical circulatory support strategies. Larger, multicenter prospective studies are warranted to confirm these results.

## Conclusions

In acute myocardial infarction–related cardiogenic shock, Impella 5+ appears to be a feasible and safe option, with a low incidence of complications. By providing higher flow rates and prolonged unloading, it can stabilize hemodynamics, optimize organ perfusion, and potentially promote native cardiac recovery. However, these encouraging results require confirmation in larger, prospective studies before definitive conclusions can be drawn.

## Data Availability

The raw data supporting the conclusions of this article will be made available by the authors, without undue reservation.
